# Supplementary material for: The selenium content of SEPP1 versus selenium requirements in vertebrates
Source: PeerJ. 2015 Sep 10;3:e1244. doi: 10.7717/peerj.1244 (PMC4699779; doi:10.7717/peerj.1244)
Supplement: Table S1 — Abbreviations; Sec, selenocysteine, SEPP1, Selenoprotein P; TXNRD, thioredoxin reductase; GPX, glutathione peroxidase; BLR, Broken line regression; Na2SeO4, sodium selenate; Na2SeO3, sodium selenite; Se-yeast, selenoyeast; NaHSeO3, sodium hydride selenite; SeMet, selenomethionine. * Methods utilised to analyse tissue GPX activity are unable to distinguish between isoforms, so are listed as total GPX activity. However, in mammals GPX1 is responsible for the majority of total GPX activity (Brigelius-Flohe et al., 2002) 1 The authors of the guinea pig study state a Se requirement of 0.08 mg Se/kg DM, which includes a safety margin above the 0.06 mg Se/kg DM predicted with BLR. 2 Data from actively growing juvenile animals was utilised in preference to adults 3 Sec content of these species were based on closely related species (Gibel carp and loach are both cyprinids, as are common carp (Cyprinus carpo) and zebrafish which both have SEPP1 (SEPP1a) with 17 Sec residues) or on salt water fish (Both green spotted pufferfish (Tetraodon nigroviridis) and fugu (Takifugu rubripes) have SEPP1 with 17 Sec residues). Irrespective of this, the range of Sec residues found in fish SEPP is small, being 15 to 17 (Lobanov, Hatfield & Gladyshev, 2008) . [file peerj-03-1244-s001.docx]

**Supplementary Table 1.** The SEPP1 Sec content, selenium requirements, and the biomarkers, statistical methods and the selenium species used to assess the selenium requirements of species included in this study.

| Class | Species | Sec in SEPP | Se require.  (mg/kg DM) | Biomarker* | Statistical method | Se species | Reference |
| --- | --- | --- | --- | --- | --- | --- | --- |
| Mammals | Guinea pig (*Cavia porcellus*)^1^ | 7 | 0.06 | Total kidney TXNRD activity | BLR | Na_2_SeO_4_ | (Jensen and Pallauf 2008) |
|  | Brown rat (*Rattus norvegicus)* | 10 | 0.10 | Total hepatic GPX activity | ANOVA | Na_2_SeO_3_ | (Weiss et al. 1996, Weiss et al. 1997) |
|  | Mouse (*Mus musculus*) | 10 | 0.10 | Total hepatic GPX activity | ANOVA | Na_2_SeO_3_ | (NRC 1995) supported by (Sunde et al. 2009) |
|  | Dog (*Canis lupus familiaris*)^2^ | 15 | 0.21 | Plasma serum Se concentration | BLR | Na_2_SeO_3_ | (Wedekind et al. 2004) |
|  | Pig (*Sus scrofa*) | 14 | 0.20 | Total GPX/GPX4 activity in heart/liver, hepatic *Gpx1/Gpx4* mRNA expression | ANOVA | Na_2_SeO_3_ | (Lei et al. 1998) |
|  | Horse (*Equus ferus caballus*) | 13 | 0.10 | Full life cycle health | - | Forage | (NRC 1997) |
|  | Cow (*Bos taurus*) | 12 | 0.10 | Full life cycle health | - | Forage | (NRC 1963) |
|  | Sheep (*Ovis aries*) | 12 | 0.10 | Full life cycle health | - | Forage | (NRC 1985) |
| Birds | Chicken (*Gallus gallus domesticus*) | 13 | 0.15 | Full life cycle health | - | Feed | (NRC 1994) |
|  | Duck (*Anas platyrhynchos*) | 13 | 0.14 | Full life cycle health | - | Feed | (NRC 1994) |
|  | Turkey (*Meleagris gallopavo*) | 13 | 0.30 | Total gizzard GPX/GPX4 activity, blood plasma GPX3 | Regression | Na_2_SeO_4_ | (Fischer et al. 2008, Sunde and Hadley 2010) |
| Bony fish | Rainbow trout (*Oncorhynchus mykiss*) | 17 | 0.30 | Blood plasma GPX3 | ANOVA | Na_2_SeO_3_ | (NRC 2011) assessed from (Hilton et al. 1980) |
|  | Channel catfish (*Ictalurus punctatus*) | 16 | 0.25 | Total hepatic GPX activity/growth | ANOVA | Na_2_SeO_3_ | (Gatlin and Wilson 1984) |
|  | Zebrafish (*Danio rerio*) | 17 | 0.30 | Growth | Regression | Se-yeast | (Penglase et al. 2014) |
|  | Loach (*Paramisgurnus dabryanus*)^3^ | 17 | 0.50 | Oxidative stress response | ANOVA | NaHSeO_3_ | (Hao et al. 2014) |
|  | Cobia (*Rachycentron canadum*) ^3^ | 17 | 0.80 | Growth and whole body/vetebrae Se retention | BLR | Se-DL-Met | (Liu et al. 2010) |
|  | Grouper (*Epinephelus malabaricus*) ^3^ | 17 | 0.70 | Growth and whole body Se retention | ANOVA and BLR | SeMet | (Lin and Shiau 2005) |
|  | Gibel carp (*Carassius auratus gibelio*) ^3^ | 17 | 1.18 | Growth | BLR | SeMet | (Han et al. 2011) |
|  | Yellowtail kingfish (*Seriola lalandi*)^3^ | 17 | 5.56 | Growth | Regression | Se-yeast | (Le and Fotedar 2013) |

Abbreviations; Sec, selenocysteine, SEPP1, Selenoprotein P; TXNRD, thioredoxin reductase; GPX, glutathione peroxidase; BLR, Broken line regression; Na_2_SeO_4_, sodium selenate; Na_2_SeO_3_, sodium selenite; Se-yeast, selenoyeast; NaHSeO_3_, sodium hydride selenite; SeMet, selenomethionine.

* Methods utilised to analyse tissue GPX activity are unable to distinguish between isoforms, so are listed as total GPX activity. However, in mammals GPX1 is responsible for the majority of total GPX activity (Brigelius-Flohe et al. 2002).

^1^The authors of the guinea pig study report a Se requirement of 0.08 mg Se/kg DM, which includes a safety margin above the 0.06 mg Se/kg DM predicted with BLR.

^2^Data from actively growing juvenile animals was utilised in preference to adults

^3^Sec content of these species were based on closely related species (Gibel carp and loach are both cyprinids, as are common carp (*Cyprinus carpo*) and zebrafish which both have SEPP1 (SEPP1a) with 17 Sec residues) or on salt water fish (Both green spotted pufferfish (Tetraodon nigroviridis) and fugu (*Takifugu rubripes*) have SEPP1 with 17 Sec residues). Overall, the number of Sec residues found in fish SEPP is 15 to 17 (Lobanov et al. 2008).

**References**

Brigelius-Flohe, R., K. Wingler and C. Muller (2002). "Estimation of Individual types of Glutathione Peroxidases." Methods in Enzymology **347**: 101-112.

Fischer, J., A. Bosse, E. Most, A. Mueller and J. Pallauf (2008). "Selenium requirement of growing male turkeys." British poultry science **49**(5): 583-591.

Gatlin, D. M., III and R. P. Wilson (1984). "Dietary selenium requirement of fingerling channel catfish." Journal of Nutrition **114**(3): 627-633.

Han, D., S. Xie, M. Liu, X. Xiao, H. Liu, X. Zhu and Y. Yang (2011). "The effects of dietary selenium on growth performances, oxidative stress and tissue selenium concentration of gibel carp (*Carassius auratus gibelio*)." Aquaculture Nutrition **17**(3): e741-e749.

Hao, X., Q. Ling and F. Hong (2014). "Effects of dietary selenium on the pathological changes and oxidative stress in loach (*Paramisgurnus dabryanus*)." Fish physiology and biochemistry.

Hilton, J. W., P. V. Hodson and S. J. Slinger (1980). "The requirement and toxicity of selenium in rainbow trout (*Salmo gairdneri*)." Journal of Nutrition **110**: 2527-2535.

Jensen, C. and J. Pallauf (2008). "Estimation of the selenium requirement of growing guinea pigs (*Cavia porcellus*)." Journal of Animal Physiology and Animal Nutrition **92**(4): 481-491.

Le, K. T. and R. Fotedar (2013). "Dietary selenium requirement of yellowtail kingfish (*Seriola lalandi*)." Agricultural Sciences **4**(6a): 68-75.

Lei, X. G., H. M. Dann, D. A. Ross, W. H. Cheng, G. F. Combs and K. R. Roneker (1998). "Dietary selenium supplementation is required to support full expression of three selenium-dependent glutathione peroxidases in various tissues of weanling pigs." The Journal of nutrition **128**(1): 130-135.

Lin, Y. H. and S. Y. Shiau (2005). "Dietary selenium requirements of juvenile grouper, *Epinephelus malabaricus*." Aquaculture **250**(1-2): 356-363.

Liu, K., X. J. Wang, Q. H. Ai, K. S. Mai and W. B. Zhang (2010). "Dietary selenium requirement for juvenile cobia, *Rachycentron canadum* L." Aquaculture Research **41**(10): e594-e601.

Lobanov, A., D. Hatfield and V. Gladyshev (2008). "Reduced reliance on the trace element selenium during evolution of mammals." Genome Biology **9**(3): R62.

NRC (1963). Nutrient requirements of beef cattle. Washington DC, National Academy Press.

NRC (1985). Nutrient requirements of sheep. Washington DC, National Academy Press.

NRC (1994). Nutrient requirements of Poultry. NRC, National Academy Press, Washington, DC.

NRC (1995). Nutrient requirements of laboratory animals. Washington DC, National Academy Press.

NRC (1997). Nutrient requirements of horses. Washington DC, National Academy Press.

NRC (2011). Nutrient requirements of fish and shrimp. Washington DC, National Academy Press.

Penglase, S., K. Hamre, J. D. Rasinger and S. Ellingsen (2014). "Selenium status affects selenoprotein expression, reproduction, and F1 generation locomotor activity in zebrafish (*Danio rerio*)." British Journal of Nutrition **111**(11): 1918-1931.

Sunde, R. A. and K. B. Hadley (2010). "Phospholipid hydroperoxide glutathione peroxidase (Gpx4) is highly regulated in male turkey poults and can be used to determine dietary selenium requirements." Experimental biology and medicine (Maywood, N.J.) **235**(1): 23-31.

Sunde, R. A., A. M. Raines, K. M. Barnes and J. K. Evenson (2009). "Selenium status highly regulates selenoprotein mRNA levels for only a subset of the selenoproteins in the selenoproteome " Bioscience Reports **29**(5): 329-338.

Wedekind, K. J., S. Yu and G. F. Combs (2004). "The selenium requirement of the puppy." Journal of Animal Physiology and Animal Nutrition **88**(9-10): 340-347.

Weiss, S. L., J. K. Evenson, K. M. Thompson and R. A. Sunde (1996). "The selenium requirement for glutathione peroxidase mRNA level is half of the selenium requirement for glutathione peroxidase activity in female rats." Journal of Nutrition **126**(9): 2260-2267.

Weiss, S. L., J. K. Evenson, K. M. Thompson and R. A. Sunde (1997). "Dietary selenium regulation of glutathione peroxidase mRNA and other selenium-dependent parameters in male rats." Journal of Nutritional Biochemistry **8**(2): 85-91.
