# Supplementary material for: The selenium content of SEPP1 versus selenium requirements in vertebrates
Source: PeerJ. 2015 Sep 10;3:e1244. doi: 10.7717/peerj.1244 (PMC4699779; doi:10.7717/peerj.1244)
Supplement: Table S2 — The total Sec (U) and the Sec content upstream and including the APOER2 binding site (E-CQC—-A; shaded in yellow) within the C-terminal domain (SEPP1←APOER2), and the region downstream of the APOER2 binding site (SEPP1APOER2→) are also shown. * Sequence obtained at ensemble.org then searched for Sec and SECIS elements using http://seblastian.crg.es/**Sequences obtained from Lobanov, Hatfield & Gladyshev (2008). Abbreviations; SEPP1←APOER2, Sec residues in the C-terminal domain of full length SEPP1in and upstream of the APOER2 binding site (E-CQC—-A; in fish may range between E-CQC–A to E-CQC—–A); SEPP1APOER2→, Sec residues in full length SEPP1upstream of the APOER2 binding site. [file peerj-03-1244-s002.docx]

**Supplementary Table 2.** The sequence ID, total number of Sec residues, and Sec residue number upstream and including the APOER2 binding site within the C-terminal domain (SEPP1^←APOER2^), and the region downstream of the APOER2 binding site (SEPP1^APOER2→^) of SEPP1 in vertebrate species included in this study and closely related species (fish).

| **Class** | **Species** | **Sequence ID** | **Total Sec** | **SEPP1 ^←APOER2^** | **SEPP1^APOER2→^** |
| --- | --- | --- | --- | --- | --- |
| **Mammals** | Human | SPP00000082_2.0 | 10 | 4 | 5 |
|  | Mouse (*Mus musculus*) | SPP00001581_2.0 | 10 | 4 | 5 |
|  | Rat (*Rattus norvegicus*) | SPP00002361_2.0 | 10 | 4 | 5 |
|  | Guinea pig (*Cavia porcellus*) | SPP00000465_2.0 | 7 | 1 | 5 |
|  | Pig (*Sus scrofa*) | SPP00002497_2.0 | 14 | 8 | 5 |
|  | Cow (*Bos taurus*) | SPP00000267_2.0 | 12 | 6 | 5 |
|  | Dog (*Canis lupus*) | SPP00000415_2.0 | 15 | 9 | 5 |
|  | Horse (*Equus caballus*) | SPP00000876_2.0 | 13 | 7 | 5 |
|  | Sheep (*Ovis aries*)** | Lobanov et al. (2008). | 12 | 6 | 5 |
| **Birds** | Chicken (*Gallus gallus*)** | Lobanov et al. (2008). | 13 | 7 | 5 |
|  | Duck (*Anas platyrhynchos*)* | ENSAPLG00000012149 | 13 | 7 | 5 |
|  | Turkey (*Meleagris gallopavo*) | SPP00001437_2.0 | 13 | 7 | 5 |
| **Fish** | Zebrafish (*Danio rerio*) | SPP00000643_2.0 | 17 | 11 | 5 |
|  | Rainbow trout (*Oncorhynchus mykiss*)** | Lobanov et al. (2008). | 17 | 11 | 5 |
|  | Channel catfish (Ictalurus punctatus)** | Lobanov et al. (2008). | 15 | 8 | 5 |
|  | Medaka (*Oryzias latipes*) | SPP00001987_2.0 | 16 | 10 | 5 |
|  | Atlantic salmon (*Salmo salar*)** | Lobanov et al. (2008). | 17 | 11 | 5 |
|  | Tilapia (*Oreochromis niloticus*) | SPP00001837_2.0 | 17 | 11 | 5 |
|  | Common carp (*Cyprinus carpio*)** | Lobanov et al. (2008). | 17 | 11 | 5 |
|  | Tetraodon (*Tetraodon nigroviridis*) | SPP00002685_2.0 | 17 | 11 | 5 |
|  | Fugu (*Takifugu rubripes*) | SPP00002593_2.0 | 17 | 11 | 5 |
|  | Cave fish (*Astyanax mexicanus*)* | ENSAMXT00000006031 | 16 | 10 | 5 |
|  | Stickleback (*Gasterosteus aculeatus*) | SPP00001102_2.0 | 15 | 9 | 5 |

*Sequence obtained at ensemble.org then searched for Sec and SECIS elements using <http://seblastian.crg.es/>

**Sequences obtained from Lobanov et al. (2008).
