# Supplementary material for: The selenium content of SEPP1 versus selenium requirements in vertebrates
Source: PeerJ. 2015 Sep 10;3:e1244. doi: 10.7717/peerj.1244 (PMC4699779; doi:10.7717/peerj.1244)
Supplement: Figure S1 — The solid lines with the solid circles (●) is the best fit model for the number of Sec residues found upstream and including the APOER2 binding site in the C-terminal of SEPP1 versus the selenium requirements (mg Se/kg DM) in mammals and bony fish. The broken lines represents the same data modelled with an additional five bony fish species with known Se requirement levels (○), but unannotated genomes as described in Fig. 2. The solid line is linear, R2 = 0.82, y = 1 + 35x, while the dashed line is 5PL asymmetric sigmoidal, R2 = 0.92, y = − 6.54 + (17.5/((1 + 10((−1.75538−X)×5.851))2.99910)). X axis is log transformed. [file peerj-03-1244-s003.docx]

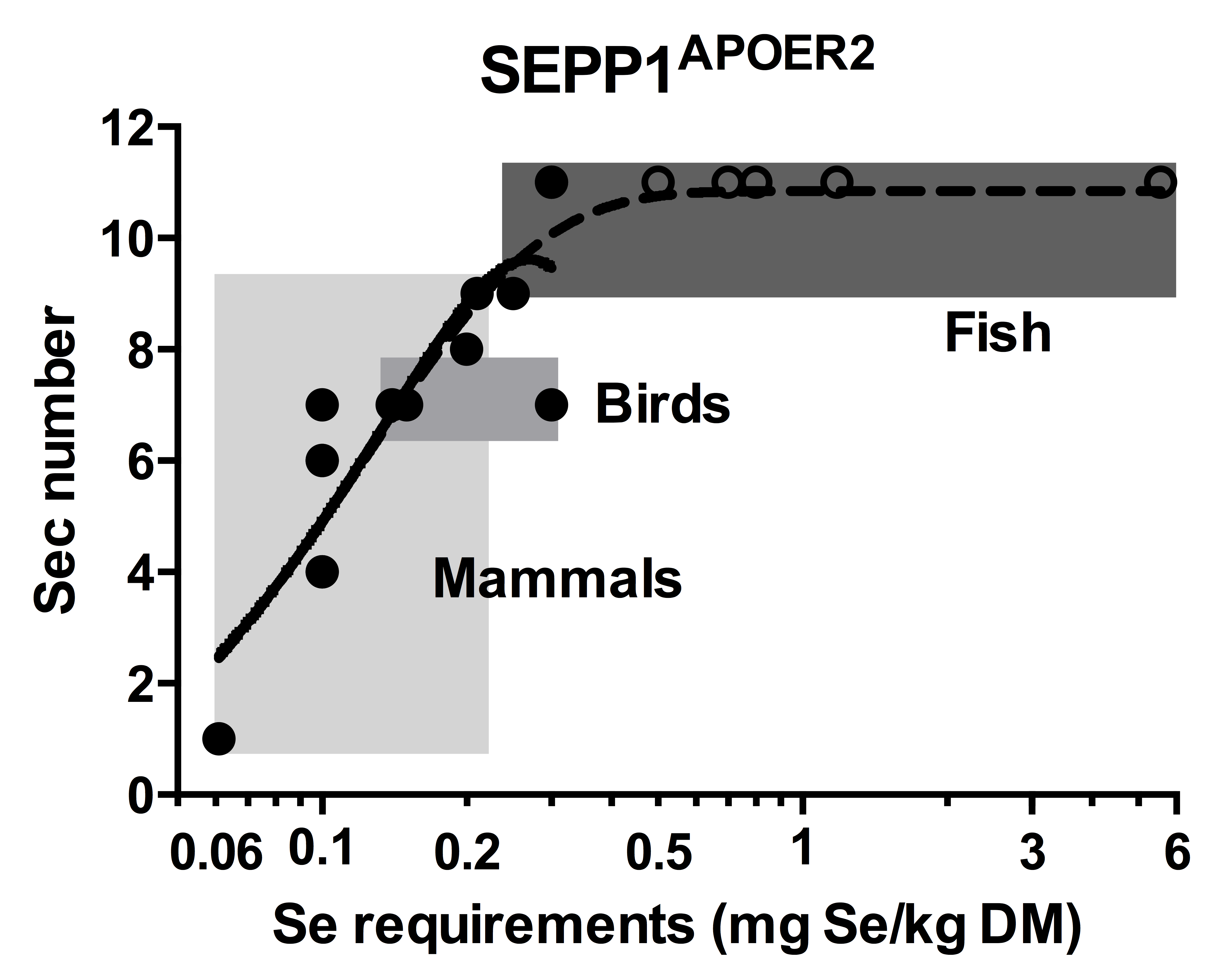


**Supplementary Figure 1.** **The relationship between the selenocysteine content within specific domains of selenoprotein P and selenium requirements.** The solid lines with the solid circles (●) is the best fit model for the number of Sec residues found upstream and including the APOER2 binding site in the C-terminal of SEPP1 versus the selenium requirements (mg Se/kg DM) in mammals, birds and bony fish. The broken lines represents the same data modelled with an additional five bony fish species with known Se requirement levels (○), but unannotated genomes as described in Fig. 2. The solid line is second order polynomial, R^2^ = 0.77, y = -2.3 + 88x - 164x^2^, while the dashed line is 5PL asymmetric sigmoidal, R^2^ = 0.86, y = -10.4 + (21.3/((1+10^(^(-2.27097-X) × 4.728^))^5.331^10^)). X axis is log transformed.
