# Supplementary material for: The selenium content of SEPP1 versus selenium requirements in vertebrates
Source: PeerJ. 2015 Sep 10;3:e1244. doi: 10.7717/peerj.1244 (PMC4699779; doi:10.7717/peerj.1244)
Supplement: Figure S2 — Multiple sequence alignment of vertebrate SEPP1. Alignment performed with Jalview (Andrew et al.2009), using MUSCLE (Robert 2004) with standard settings. Sequences not listed in Table S2 were obtained from the selenoprotein database (selenodb.com, (Romagné et al., 2014)). MUSCLE does not recognize the amino acid symbol for selenocystiene (u), replacing u with x during alignment analysis. All x’s within the sequences represent selenocysteine residues. Animals included in the manuscript are provided in the top list of sequences, followed by the invertebrate Lottia gigantean sequence (Liang, Jiazuan & Qiong, 2012) used as an outgroup for building a phylogenetic tree, additional mammal sequences available on selenoDB (version 2.0) or the naked mole rat (Heterocephalus glaber, XP_004848622.1) genome database (http://www.naked-mole-rat.org/). [file peerj-03-1244-s004.docx]

**Supplementary Figure 2**. Alignments of vertebrate SEPP1.


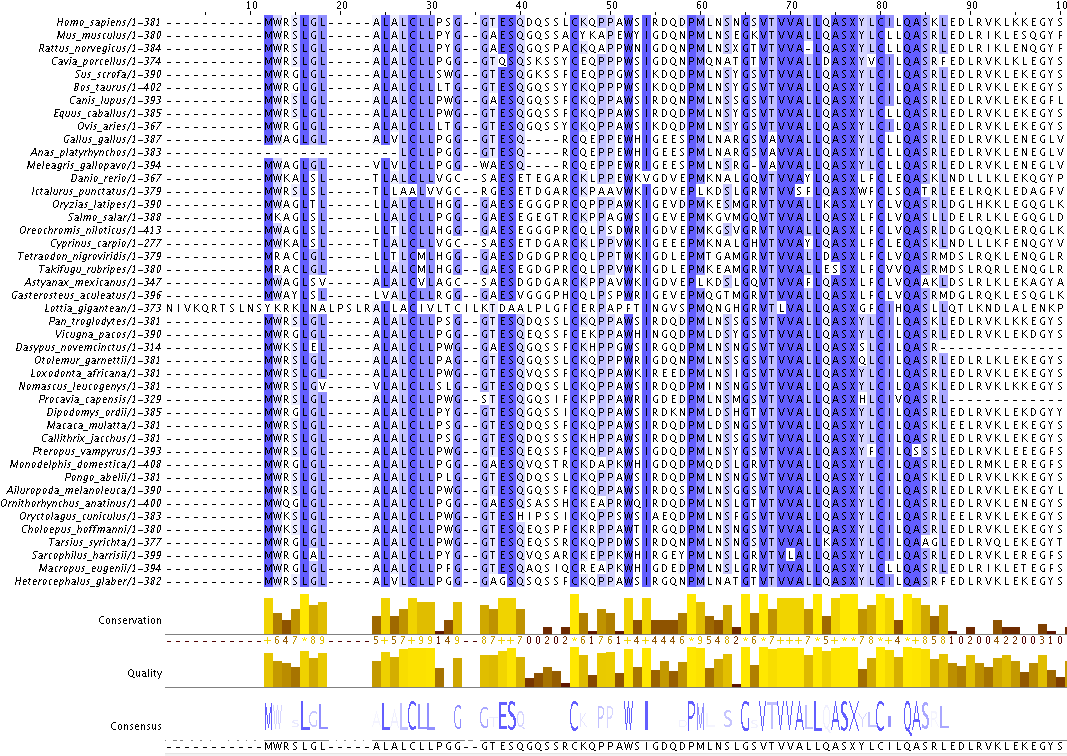


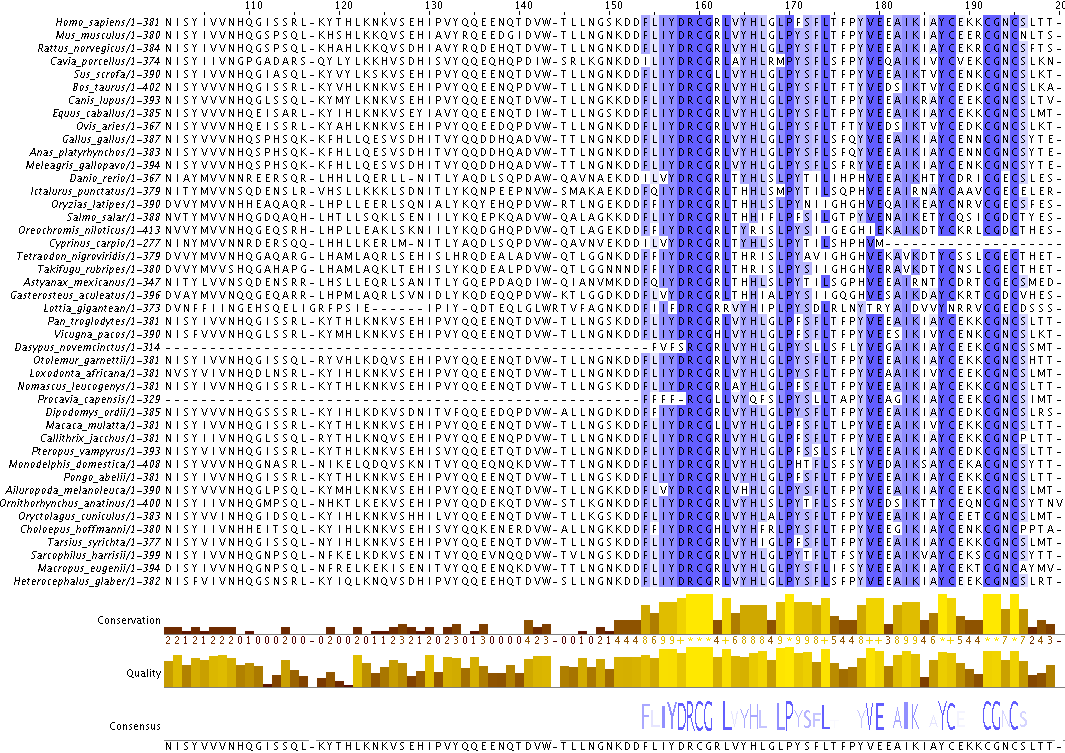


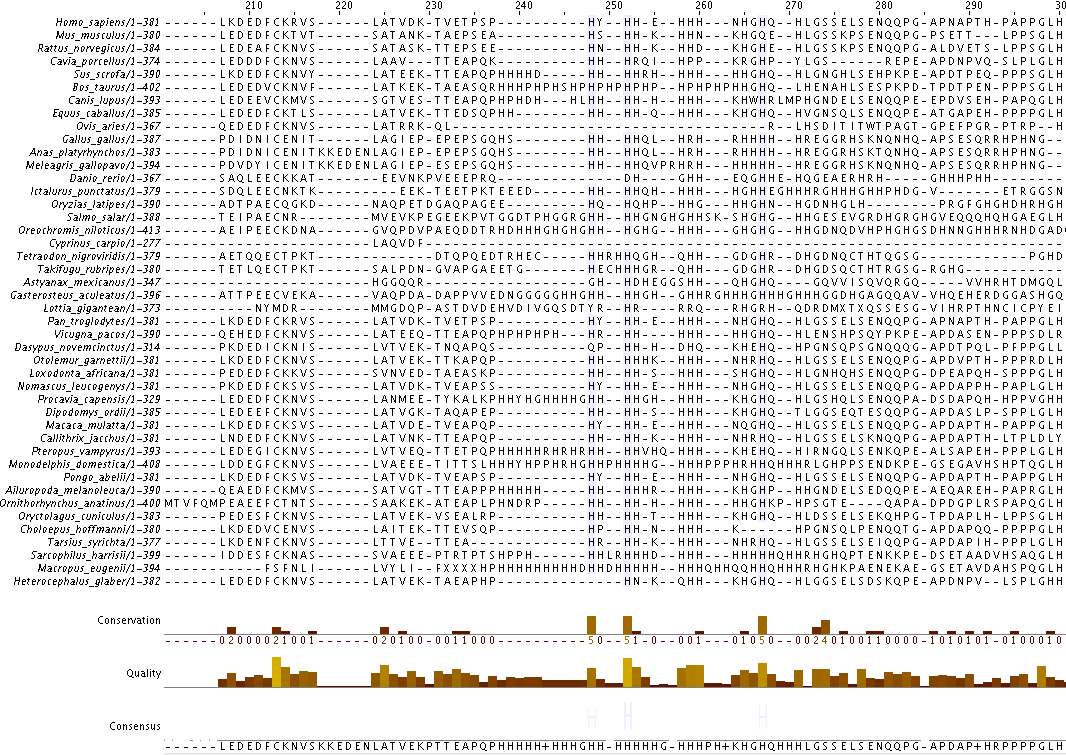


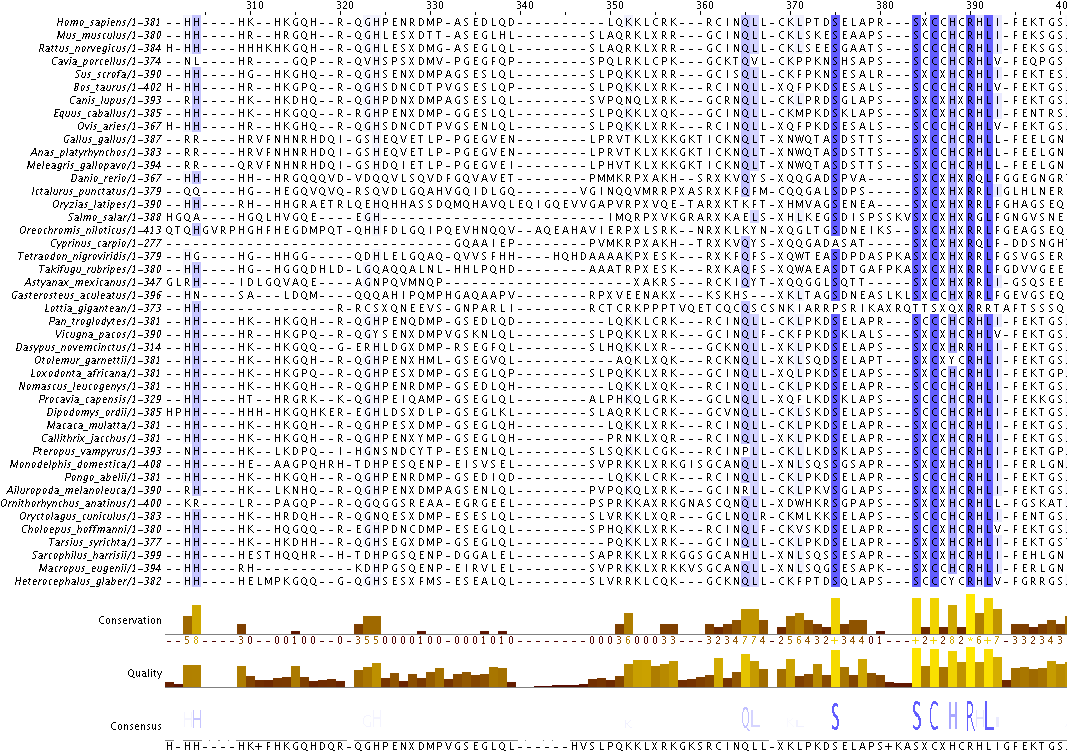


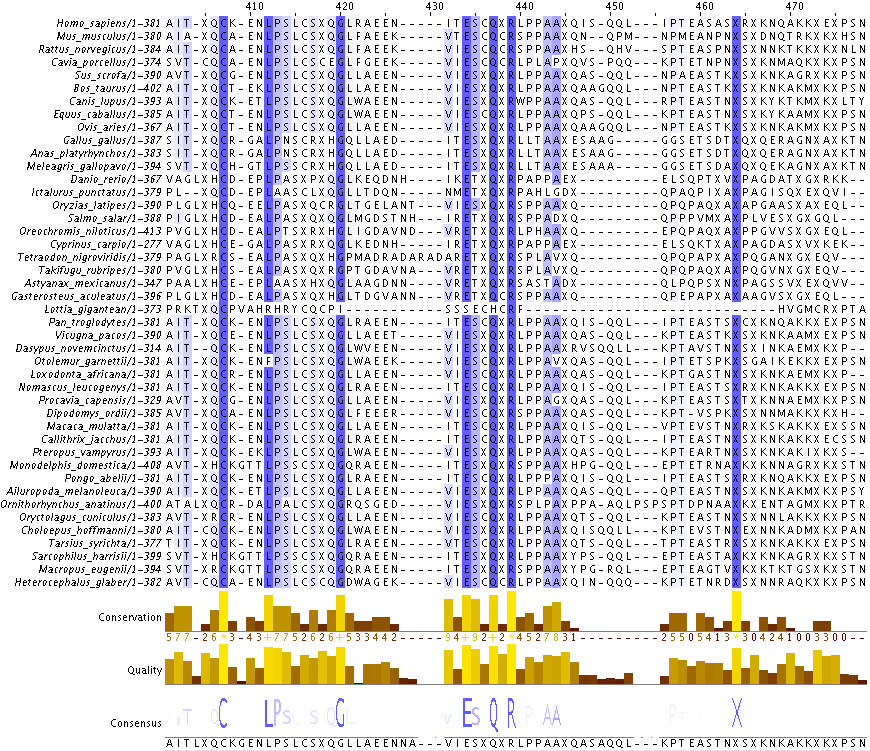


**Supplementary Figure 2**. **Alignments of vertebrate SEPP1.**

Multiple sequence alignment performed with Jalview (Andrew et al. 2009), using MUSCLE (Robert 2004) with standard settings. Sequences not listed in Supplementary Table 2 were obtained from the selenoprotein database (selenodb.com, (Romagné et al. 2014)). MUSCLE does not recognize the amino acid symbol for selenocystiene (u), replacing u with x during alignment analysis. All x’s within the sequences represent selenocysteine residues. Animals included in the manuscript are provided in the top list of sequences, followed by the invertebrate *Lottia gigantean* sequence (Liang et al. 2012) used as an outgroup for building a phylogenetic tree, additional mammal sequences available on selenoDB (version 2.0) or the naked mole rat (*Heterocephalus glaber,* XP_004848622.1) genome database (http://www.naked-mole-rat.org/).

Andrew, M. W., B. P. James, M. A. M. David, C. Michèle and J. B. Geoffrey (2009). "Jalview Version 2—a multiple sequence alignment editor and analysis workbench." Bioinformatics.

Liang, J., N. Jiazuan and L. Qiong (2012). "Evolution of selenoproteins in the metazoan." BMC Genomics **13**(1): 446.

Robert, C. E. (2004). "MUSCLE: multiple sequence alignment with high accuracy and high throughput." Nucleic Acids Research.

Romagné, F., D. Santesmasses, L. White, G. K. Sarangi, M. Mariotti, R. Hübler, A. Weihmann, G. Parra, V. N. Gladyshev, R. Guigó and S. Castellano (2014). "SelenoDB 2.0: annotation of selenoprotein genes in animals and their genetic diversity in humans." Nucleic acids research **42**(Database issue): D437-443.
