# Supplementary material for: The selenium content of SEPP1 versus selenium requirements in vertebrates
Source: PeerJ. 2015 Sep 10;3:e1244. doi: 10.7717/peerj.1244 (PMC4699779; doi:10.7717/peerj.1244)
Supplement: Figure S3 — A UPGMA tree is displayed. Support values shown are the UPGMA bootstrap values. The tree was configured using standard settings in MEGA Ver. 6.06 (Koichiro et al.2013). Sequences were obtained as described in Fig. S3. Lottia gigantean was utilised as a non-vertebrate outgroup. [file peerj-03-1244-s005.docx]

**Supplementary Figure 3.** **Phylogenetic tree of vertebrate selenoprotein P.** A UPGMA tree is displayed. Support values shown are the UPGMA bootstrap values. The tree was configured using standard settings in MEGA Ver. 6.06 (Koichiro et al. 2013). Sequences were obtained as described in Supp. Fig. 3. *Lottia gigantean* was utilised as a non-vertebrate outgroup.

**References**

Koichiro, T., S. Glen, P. Daniel, F. Alan and K. Sudhir (2013). "MEGA6: Molecular Evolutionary Genetics Analysis Version 6.0." Molecular Biology and Evolution.
